# Potential efficacy and preliminary mechanistic insights of the Jianpi Yishen Zhuanggu Tongluo formula for rheumatoid arthritis with sarcopenia-osteopenia: an integrated pilot study

**DOI:** 10.3389/fphar.2026.1756789

**Published:** 2026-04-21

**Authors:** Weidong Liang, Liuting Chen, Yadan Zhang, Jianhong Peng

**Affiliations:** 1 Department of Rheumatology, Dongguan Hospital of Traditional Chinese Medicine, Dongguan, China; 2 Department of Rheumatology, Guangzhou University of Chinese Medicine Dongguan Hospital, Dongguan, China

**Keywords:** rheumatoid arthritis with sarcopenia-osteopenia, Jianpi Yishen Zhuanggu Tongluo therapy, pilot study, metabolomics, mitochondrion

## Abstract

**Objectives:**

To explore the potential efficacy and identify preliminary metabolic biomarkers of the Jianpi Yishen Zhuanggu Tongluo formula (JPYSZGT) in rheumatoid arthritis (RA) complicated with sarcopenia-osteopenia (SO) using 1H-NMR-based metabolomics and *in vitro* experiments.

**Methods:**

In this pilot study, forty RA-SO patients were randomly allocated into a treatment group (Group C, n = 20) receiving JPYSZGT plus conventional therapy and a control group (Group D, n = 20) receiving conventional therapy alone for 12 months. Clinical outcomes, including inflammatory markers (CRP, ESR), bone metabolism markers (CTX, PINP), insulin-like growth factor (IGF), cytokines (IL-6, TNF-α), bone mineral density (BMD), and appendicular skeletal muscle mass index (ASMI), were analyzed.

**Results:**

Compared to Group D, Group C showed greater improvements in DAS28-ESR, ASMI, and BMD, and greater reductions in ESR, CRP, TNF-α, CTX, and PINP, along with a significant increase in IGF levels. Metabolomics analysis identified preliminary alterations in 14 pathways involving glucose, lipid, and amino acid metabolisms. *In vitro* experiments suggested that JPYSZGT may suppress macrophage activation, potentially upregulate PINK1/PARKIN, downregulate OPG/RANK pathways, and reduce TNF-α and IL-6 secretion.

**Conclusion:**

This exploratory study suggests that JPYSZGT may improve bone-muscle metrics in RA-SO and that its potential mechanisms could involve the modulation of the PINK1/PARKIN, OPG/RANK, and PI3K/Akt pathways. It also appears to inhibit macrophage-driven inflammation. These findings warrant further investigation in larger-scale studies.

**Clinical Trial Registration:**

identifier ChiCTR2500106729.

## 1 Introduction

Rheumatoid arthritis (RA) is an immune-mediated disorder that features chronic synovitis. It presents with morning stiffness, joint pain, and erosive polyarthritis caused by progressive and destructive inflammation (Tian et al., 2025). Osteoporosis (OP) and sarcopenia are highly prevalent comorbidities in RA, creating a complex clinical triad—RA with sarcopenia-osteopenia (RA-SO)—that severely impacts patient quality of life and functional capacity (Buehring et al., 2021; Chen et al., 2020). Systemic bone loss in RA often manifests early (Richards et al., 2025), even preceding clinical symptoms. RA profoundly increases the risk of osteoporosis, resulting in a debilitating comorbidity that escalates patient suffering and healthcare costs. Recent evidence confirms a high prevalence, with a 2022 meta-analysis reporting osteoporosis in 46.1% of RA patients, which is driven by chronic inflammation and treatments that accelerate bone loss (Minjie et al., 2023). This synergistic effect doubles the risk of major osteoporotic fractures compared to the general population, leading to severe pain, disability, and loss of independence (Ramos et al., 2025). The resulting cycle of functional decline and increased care needs imposes a substantial burden on both individuals and healthcare systems, underscoring an urgent need for proactive, integrated management strategies in clinical practice (Li et al., 2025). Activated macrophages in RA drive systemic inflammation and serve as pivotal mediators of bone resorption and erosion (Liu et al., 2025), which makes macrophage polarization a promising therapeutic target.

Current management of RA-SO includes disease-modifying therapies combined with bone-protective agents like bisphosphonates and resistance training. However, these conventional approaches often provide incomplete protection for bone and muscle, and long-term use of certain pharmaceuticals is associated with potential adverse effects. JPYSZGT is administered orally as a decoction (200 mL twice daily) and comprises 12 botanical ingredients formulated to tonify the spleen and kidney, to strengthen the bones, and to promote blood circulation. Other traditional Chinese medicine interventions, such as Tai Chi and Baduanjin exercises, have also shown benefits in improving physical function and reducing pain in patients with RA or osteoporosis (Wu et al., 2023; Yang et al., 2025). Compared to these mind-body modalities, JPYSZGT represents a pharmacotherapeutic approach targeting systemic bone-muscle metabolism. Recent research highlights the potential of traditional Chinese medicine (TCM) formulas that tonify the kidney and strengthen the spleen (Lin et al., 2025; Gu et al., 2024). These approaches aim to modulate inflammation and bone metabolism simultaneously, offering a promising integrated strategy for managing this dual burden.

Despite its clinical use, the scientific evidence base for JPYSZGT, particularly concerning its effect on the bone-muscle axis in RA-SO, remains limited. A comprehensive understanding of its potential mechanisms is lacking. To address this gap, we adopted an integrated research strategy. This pilot study combines a clinical trial with 1H-NMR-based metabolomics and *in vitro* experiments to explore the potential effects of JPYSZGT and generate hypotheses regarding its mechanisms of action.

Therefore, the primary objective of this exploratory study was not to provide definitive proof of efficacy, but to conduct an initial investigation into the potential of JPYSZGT for RA-SO. We aimed to preliminarily assess its impact on clinical outcomes, identify potential metabolic biomarkers, and explore its possible mechanisms related to macrophage polarization, mitophagy (PINK1/PARKIN pathway), and bone metabolism (OPG/RANK and PI3K/Akt pathways). We posit that this integrative, hypothesis-generating approach will provide a valuable foundation for future large-scale, confirmatory trials.

## 2 Subjects and reagents

### 2.1 Study population and sample size consideration

A total of 40 hospitalized patients diagnosed with rheumatoid arthritis (RA) complicated by sarcopenia and osteopenia (SO) were recruited from Dongguan Hospital of Traditional Chinese Medicine from July 2022 to February 2024.

This study was approved by the Ethics Committee of Dongguan Hospital of Traditional Chinese Medicine (Approval number PJ [2022] No.92 and PJ[2024] No.29.) on March 2022. All participants provided written informed consent prior to enrollment. The study was conducted from July 2022 to February 2024.

Given the exploratory nature of this pilot study and the challenges in recruiting patients with the specific comorb RA-SO, a formal *a priori* sample size calculation was not performed. The sample size of 40 participants was determined based on feasibility and patient availability during the predefined recruitment. sample size is comparable to those used in previous pilot and preliminary studies in the field of TCM for complex rheumatic conditions (Zhang et al., 2024).

### 2.2 Study design and outcome measures

This was a single-center, exploratory, randomized study. The change from baseline in bone mineral density (BMD) was pre-specified as the primary clinical outcome for assessing efficacy. Changes in 28 joints and erythrocyte sedimentation rate (DAS28-ESR), appendicular skeletal muscle mass index (ASMI), serum biomarkers (including inflammatory cytokines and bone metabolism markers), and metabolomic profiles were considered exploratory secondary outcomes.

### 2.3 Diagnostic criteria

Participants were required to meet both Western medicine and Traditional Chinese Medicine (TCM) diagnostic criteria for enrollment.

#### 2.3.1 Western Medicine diagnostic criteria

Rheumatoid arthritis (RA) was diagnosed according to the 1987 American College of Rheumatology (ACR) or the 2010 ACR/European League Against Rheumatism (EULAR) classification criteria (score ≥6), with exclusion of other autoimmune rheumatic diseases. Osteoporosis (OP) was diagnosed based on the guidelines of the Chinese Society of Osteoporosis and Bone Mineral Research, defined either by the presence of fragility fractures occurring without significant trauma or by a Dual-energy X-ray absorptiometry (DXA) T-score of less than −1.0 standard deviation (SD), indicating osteopenia or osteoporosis. Sarcopenia was defined according to the Asian Working Group for Sarcopenia (AWGS) 2019 consensus, using either an appendicular skeletal muscle mass index (SMI) less than 2 SD below the mean for young adults of the same ethnic group, or bioelectrical impedance analysis (BIA) thresholds of <7.0 kg/m^2^ for men and <5.7 kg/m^2^ for women.

The TCM syndrome pattern was identified as Spleen-Kidney Yang Deficiency with Blood Stasis, based on the following criteria from established guidelines (2019 Guideline for Osteoporosis Integrated TCM-Western Medicine; 2020 TCM Guideline for Rheumatoid Arthritis; GB/T16751.2–2021). Primary symptoms included fixed joint pain with tenderness, cold pain in the lower back and knees, poor appetite with loose stools, and limb weakness. Secondary symptoms encompassed cold intolerance, abdominal distension, clear and profuse urine, and a sallow or dull complexion. Tongue and pulse characteristics required for diagnosis were a dark-red tongue with ecchymosis and a thin or thick white coating, accompanied by a deep, wiry, or thready-choppy pulse.

### 2.4 Inclusion criteria

Participants were eligible for inclusion if they met all of the following criteria: (a) fulfillment of both the Western and TCM diagnostic criteria described above; (b) age between 18 and 75 years; (c) willingness and ability to comply with the treatment and follow-up protocols; and (d) provision of written informed consent prior to study participation.

### 2.5 Exclusion criteria

Individuals were excluded from the study if they met any of the following conditions: (a) pregnancy or lactation; (b) severe malnutrition; (c) major comorbidities involving the cardiac, cerebral, renal, or hematological systems; (d) concurrent other rheumatic diseases; (e) known allergy to any botanical drug contained in the JPYSZGT formulation; or (f) inability to comply with the study procedures.

### 2.6 Withdrawal criteria

Withdrawal criteria include non-adherence to treatment, deviations from the protocol such as switching therapies, or severe adverse events that require discontinuation.

### 2.7 Group allocation

Following enrollment, eligible participants were randomly assigned to the treatment group (Group C) or the control group (Group D) using a computer-generated random number table, with 20 patients in each group. The allocation sequence was concealed in sequentially numbered, opaque, sealed envelopes to ensure proper randomization.

Patients in both groups received standard conventional Western medical treatment throughout the 12-month study period. This background therapy consisted of methotrexate (10 mg orally once weekly) and a combination of alendronate and vitamin D (70 mg orally once weekly), administered according to standard clinical protocols for rheumatoid arthritis with osteoporosis.

In addition to this conventional therapy, patients in the treatment group (Group C) were administered the Jianpi Yishen Zhuanggu Tongluo (JPYSZGT) formula. The JPYSZGT decoction was prepared daily as described in Section 2.9 and administered orally at a dose of 200 mL twice daily (morning and evening). Patients in the control group (Group D) received the conventional therapy alone, without any additional herbal intervention.

All medications were self-administered by patients at home, with compliance monitored through monthly follow-up visits and medication diaries. Treatment adherence and any adverse events were documented throughout the 12-month intervention period.

### 2.8 Plant materials and authentication

The Jianpi Yishen Zhuanggu Tongluo formula (JPYSZGT) is a decoction composed of 12 medicinal plants. All botanical materials were sourced from the certified pharmacy of Dongguan Hospital of Traditional Chinese Medicine, which procures them from suppliers compliant with Good Agricultural and Collection Practices (GACP). The botanical identification of all crude drugs was performed by Deputy Chief Pharmacist Zhiming Mo of our institution. Identification was conducted with reference to the Pharmacopoeia of the People’s Republic of China (2020 edition) and verified using the Plants of the World Online (POWO) database. Voucher specimens for each batch of crude drugs have been deposited in the Herbarium of Dongguan Hospital of Traditional Chinese Medicine (Voucher specimen numbers: DGCM-Herb-2022-JPYSZGT-01–12). None of the species used are listed as endangered or restricted under the Convention on International Trade in Endangered Species of Wild Fauna and Flora (CITES).

The complete formulation, with standardized botanical nomenclature (including family, genus, species, authority, and plant part used), is detailed in Table 1.

**TABLE 1:** Composition and botanical authentication of the JPYSZGT formula.

No.	Pharmaceutical name (Chinese)	Dosage (g per daily decoction	Scientific name (family)	Plant part used	Voucher specimen number
1	Du Zhong	15	Eucommia ulmoides Oliv. [Eucommiaceae]	Bark	DDZ-20220701
2	Xu Duan	15	Dipsacus asperoides C.Y.Cheng and T.M.Ai [Caprifoliaceae]	Root	DXDU-20220702
3	Tu Si Zi	15	Cuscuta chinensis Lam. [Convolvulaceae]	Seed	DTSZ-20220703
4	Huang Qi	15	Astragalus membranaceus (Fisch.) Bunge [Fabaceae]	Root	DHQ-20220704
5	Wu Wei Zi	6	Schisandra chinensis (Turcz.) Baill. [Schisandraceae]	Fruit	DWWZ-20220705
6	Fu Ling	12	Poria cocos (Schw.) Wolf [Polyporaceae]	Sclerotium	DFL-20220706
7	Dang Gui	12	Angelica sinensis (Oliv.)Diels [Apiaceae]	Root	DDG-20220707
8	Niu Xi	12	Achyranthes bidentata Blume [Amaranthaceae]	Root	DNX-20220708
9	Lu Jiao Jiao	10	Cervus nippon Temminck [Cervidae]	Colla (gelatin)	DLJJ-20220709
10	Tao Ren	10	Prunus persica (L.) Batsch [Rosaceae]	Seed	DTR-20220710
11	Gu Sui Bu	10	Drynaria roosii Nakaike [Polypodiaceae]	Rhizome	DGSB-20220711
12	Shu Di Huang	10	Rehmannia glutinosa (Gaertn.) DC. [Orobanchaceae]	Prepared Root	DSDH-20220712

Cervi Cornus Colla (Lu Jiao Jiao): This ingredient is an animal-derived traditional medicine. It from the antler of the Sika deer (Cervus nippon Temminck) or the Red deer (*Cervus elaphus* Linnaeus. specific product used in this study was obtained from a GMP-certified supplier (Dong’e Ajiao Co., Ltd., China Pharmaceutical Product Registration No.: [国药准字Z65020137), ensuring it was sourced from farmed deer and not from endangered or wild populations. Its use complies with the Pharmacopoeia of the’s Republic China (2020 edition) and relevant Chinese regulations for traditional medicines.

### 2.9 Preparation of the JPYSZGT decoction

The decoction was prepared daily in the hospital’s pharmacy according to a standardized protocol. The constituent herbs in the proportions listed in Table 1 were immersed in 1 L of distilled water for 30 min, then boiled and simmered for 40 min. The extract was filtered, and the process was repeated once by adding more water to the residue. The two filtrates were combined and concentrated under reduced pressure to a final concentration equivalent to 2.0 g of raw herbal material per mL (drug-to-extract ratio: 1:2). This concentrated decoction was stored at 4 °C and provided to patients in the treatment group for daily oral administration. Patients in the treatment group received 200 mL of the JPYSZGT decoction orally, twice daily (morning and evening), for 12 consecutive months.

### 2.10 UPLC-Q-TOF-MS analysis and data processing

Chemical profiling of JPYSZGT was performed using UPLC-Q-TOF-MS (Thermo Fisher Scientific). Chromatographic separation used a HSS T3 column with 0.1% formic acid in water/acetonitrile gradient elution. MS detection operated in positive/negative ionization modes with full MS (m/z 100–1500) and data-dependent MS/MS acquisition.

Raw data were processed using Xcalibur 4.1 software. The workflow included: (1) Automated peak detection and alignment using the integrated Compound Discoverer module with 5 ppm mass tolerance; (2) Background subtraction using blank samples; (3) Tentative compound identification by matching accurate mass (<5 ppm) and MS/MS spectra against HMDB and mzCloud databases, with verification using reference standards.

This standardized protocol ensured reliable characterization of JPYSZGT constituents, providing a reproducible chemical fingerprint for quality assessment.

### 2.11 Experimental materials

Fetal bovine serum (FBS, Gibco, USA); 0.25% trypsin (Sigma-Aldrich, USA); penicillin-streptomycin solution (Beyotime Biotechnology, China); DMEM medium (Gibco, USA); PBS buffer (Datasheer, China); Rat Bone Marrow Mesenchymal Stem Cell Osteogenic Differentiation Kit (Cyagen, China); 4% paraformaldehyde solution (Beyotime Biotechnology, China); TRAP/ALP Double Staining Kit (Fujifilm, Japan); Alizarin Red Staining Solution (pH 5.1–5.3, Cyagen, China); Red Blood Cell Lysis Buffer (Boster Biological, China); anhydrous sodium dihydrogen phosphate (NaH_2_PO_4_, Guangshi Co., Ltd., China); disodium hydrogen phosphate dihydrate (Na_2_HPO_4_·2H_2_O, Yueqiao Co., Ltd., China); deuterium oxide (D_2_O, Hangpu Laboratory Equipment Co., Ltd., China).

The RAW264.7 cells used in this study were purchased from Procell Life Science & Technology Co., Ltd., China.

### 2.12 Experimental instruments

Microplate Oscillator (Jierui’an Instruments Equipment Co., Ltd.); Nuclear Magnetic Resonance (NMR) Tube (Pu Experimental Supplies Co., Ltd.); TGL-20M Benchtop High-Speed Refrigerated Centrifuge (Luxiangyi Centrifuge Instrument Co., Ltd.); Superconducting Nuclear Magnetic Resonance Spectrometer (Varian Co., Ltd.); X-ray Bone Densitometer (GE Healthcare Systems Ultrasound and Basic Medical Diagnostics Co., Ltd.).

## 3 Experiment and methods

### 3.1 Sample collection

#### 3.1.1 Score scale

This project uses the VAS score, DSA-ESR, and bioelectric impedance methods to assess limb skeletal muscle quality. The research subjects were questioned and tested face to face by a designated physician, and the questionnaire was completed based on their responses.

#### 3.1.2 Laboratory inspection

Measurement of islet-derived factors, vitamin D, bone metabolism markers, and inflammatory cytokines (ESR, CRP, TNF-α, IL-6) was performed using ELISA in patients with rheumatoid arthritis and sarcopenia-osteoporosis before and after treatment.

#### 3.1.3 Bone density detection

Dual energy X-ray was used to measure bone density before and after treatment in patients with sarcosmic osteoporosis. The T values of the lumbar spine, hip, and femur were recorded separately.

#### 3.1.4 Sample pretreatment

6 mL of blood samples were collected from patients and healthy subjects, then centrifuged for 15 min at 4 °C with a speed of 10,000 rpm. Then, 300 μL of the supernatant serum was transferred into an NMR tube, followed by the addition of 200 μL of 0.2 mol/L phosphate buffer solution (pH = 7.4) and 50 μL of deuterium oxide (D2O), and the mixture was vortexed thoroughly.

#### 3.1.5 NMR experiment

At 25 °C, the pulse sequence was set to Carr-Purcell-Meiboom-Gill, the spectral width was 10,000 Hz, the echo time (TE) was 1 m, and 64k data points, 64 cycles, and 128 scans were performed. The nuclear magnetic resonance signal was converted into a spectrum through Fourier transformation.

#### 3.1.6 Graph processing and metabolomic data analysis

We used the professional software MestReNova (version 8.0.1) to analyze the map, setting the lactate peak (δ 1.33) as the chemical shift reference, as described in standard metabolomics protocols (Dona et al., 2016). The integral data were normalized, saved in Excel, and subjected to further multivariate statistical analysis.

The normalized integral data were imported into SIMCA-P+ 14.0 software (Umetrics, Sweden) for multivariate statistical analysis, including unsupervised Principal Component Analysis (PCA) and supervised Partial Least Squares-Discriminant Analysis (PLS-DA) and Orthogonal PLS-DA (OPLS-DA). Model quality was assessed by the parameters R2 (goodness of fit) and Q2 (goodness of prediction).

#### 3.1.7 Statistical analysis for clinical and metabolomic data

For the analysis of NMR-based metabolomic data, metabolites with a variable importance in projection (VIP) score ≥1.0 from the OPLS-DA model and a p-value <0.05 from univariate tests (see below) were considered differentially expressed. To address the multiple comparisons problem inherent in untargeted metabolomics, the false discovery rate (FDR) was calculated using the Benjamini–Hochberg method. Metabolites with an FDR-corrected q-value <0.2 were highlighted as being of particular interest.

For other experimental data, the Shapiro-Wilk test was used to assess normality of continuous variables. If the data met the normal distribution, the mean ± standard deviation is used for statistical description. Differences between groups were compared using independent sample t-test, and paired t-test was used for pre- and post-comparison. If the data did not meet the normal distribution, the median (interquartile range) is used for statistical description. The non-parametric Mann-Whitney U test was used for comparison between groups, and the paired Wilcoxon test was used for pre- and post-comparison. Categorical variables were statistically described using the number of cases (proportion %), and differences were compared by the χ^2^ test. Statistical analysis was performed using SPSS 25.0 and R 4.2.0, and the test level was α = 0.05.

### 3.2 *In vitro* experiments

#### 3.2.1 Cell culture

Culture of macrophages (RAW264.7): Cells were seeded in culture medium containing M-CSF and 50 ng/mL RANKL. The culture dish was incubated at 37 °C with 5% CO_2_, and the culture medium was replaced every 2–3 days. After incubation for 3–10 passages, the cells were used for subsequent experiments.

#### 3.2.2 Drug preparation

Prepare the Chinese medicine decoction 1, then concentrate the decoction to a solution containing 2 g/mL of raw medicine using a rotary evaporator; place it at −80 °C for 72 h; then subject it to vacuum freeze-drying. After drying, collect the powder into a 50 mL centrifuge tube, with a net weight of 63.788 g, and store it at 4 °C. Dissolve the lyophilized powder in DMEM containing 10% FBS and 1% β-mercaptoethanol, and prepare a medicinal solution at a concentration of 10 mg/mL. Filter sterilize the solution using a 0.22 μm filter membrane, aliquot it, and store at −20 °C until use.

#### 3.2.3 CCK-8 detects proliferation of RAW264.7

CCK-8 was used to observe the effects of different concentrations of Chinese medicine lyophilized powder and alendronate on the proliferation of RAW264.7 cells. The maximum safe concentration of these compounds was also determined. RAW264.7 cells were seeded on 96-well plates, with three duplicate wells set in each group, at a density of 0.5 × 10^4 cells per well, and cultured in DMEM medium containing 10% FBS and 1% antibodies. After 1 day of culture, cells were treated with different concentrations of Chinese medicine lyophilized powder (0, 25, 50, 100, 200, 400, 600, 800, and 1000 μg/L), sodium alendronate (10 μg/mL), or zoledronic acid (1 μg/mL) for 24, 48, and 96 h. Then, 10 μL of CCK-8 reagent was added per well and incubated for 70 min at 37 °C. The absorbance (optical density, OD) was measured at 450 nm using a microplate reader. The cell survival rate of each group was calculated using the formula: cell survival rate = (OD experimental well − OD blank well)/(OD negative control − OD blank well).

#### 3.2.4 Determination of expression of parkin, LC3B, p-p65, p-IKKα/β, IκBα, and RANKL proteins in each group of macrophages

Following removal of culture medium, adherent cells were washed twice with PBS. Cell lysis was performed using ice-cold lysis buffer for 15 min at 4 °C. Lysates were centrifuged at 12,000 rpm for 15 min to pellet debris. Total cellular protein in the supernatant was quantified using the BCA method according to the manufacturer’s specifications. Equal protein amounts were denatured in SDS loading buffer at 95 °C for 5 min, then separated by SDS-PAGE electrophoresis at 110 V. Proteins were subsequently transferred to PVDF membranes. After blocking with 5% skim milk in TBST for 2 h at room temperature, membranes were sectioned by molecular weight and probed overnight at 4 °C with rabbit-derived primary antibodies against parkin, LC3B, p-p65, p-IKKα/β, IκBα, and RANKL (1:1500 dilution). Membranes were washed extensively with TBST and incubated with HRP-conjugated sheep anti-rabbit secondary antibody (1:2000) for 1 h at room temperature. Following additional TBST washes, protein bands were visualized using enhanced chemiluminescence and imaged digitally. Quantitative analysis was performed using Quantity One software to determine relative protein expression levels.

#### 3.2.5 ELISA method to detect TNF-α, IL-1, IL-6 and IL-17 content in each group

After equilibrating at room temperature for 60 min, remove necessary microplate strips from the foil bag, seal the remaining strips in a storage bag, and return to 4 °C. Place strips in the frame, designating wells for standards, samples, and blanks. Add 50 μL of each standard to the respective wells and 50 μL of each sample to its wells, leaving blank wells empty. Add 100 μL of HRP-conjugated detection antibody to all wells except the blanks. Seal the plate and incubate at 37 °C for 60 min. Discard the liquid and wash all wells five times with 350 μL wash buffer, waiting 1 minute each time before drying thoroughly. Add 50 μL of substrate solution A and then 50 μL of substrate solution B to every well. Seal the plate and incubate protected from light at 37 °C for 15 min. Add 50 μL of stop solution to every well and measure optical density at 450 nm within 15 min.

#### 3.2.6 Statistical method

The integral data were subjected to multivariate analysis using SIMCA-P+ 14.0 software. Principal component analysis (PCA), partial least squares-discriminant analysis (PLS-DA), and orthogonal PLS-DA (OPLS-DA) were performed. Model validity was assessed based on R^2^ (goodness of fit) and Q^2^ (predictive ability) values, with both exceeding 0.5 considered acceptable. Score plots derived from OPLS-DA visualized group separation, while loading plots identified spectral features contributing to class discrimination. Differential chemical shifts observed in score plots were cross-referenced with one-dimensional ^1^H-NMR spectra to assign corresponding metabolites.

For conventional statistical analysis, continuous variables were tested for normality using the Shapiro–Wilk test. Normally distributed data are presented as mean ± standard deviation and were compared using the independent samples t-test (between groups) or paired t-test (within groups). Non-normally distributed data are expressed as median (interquartile range) and analyzed with the Mann–Whitney U test (between groups) or Wilcoxon signed-rank test (within groups). Categorical variables are summarized as number (percentage) and compared using the χ^2^ test. All analyses were performed with SPSS 25.0 and R 4.2.0, with a two-sided α level of 0.05 considered statistically significant.

## 4 Experimental results

### 4.1 General information

The baselines of the two groups were consistent in terms of age and gender, and there were no statistical differences, as shown in Table 2.

**TABLE 2:** Comparison of age and gender between the two groups.

Group	Number of cases (n = 40)	Gender (Male, n (%))	Gender (Female, n (%))	Age (years, mean ± SD)
Treatment group	20	3 (15.00)	17 (85.00)	65.25 ± 8.75
Control group	20	3 (15.00)	17 (85.00)	67.50 ± 7.16
χ^2^/t	​	0.000		−0.890
P	​	1.000		0.379

### 4.2 Changes in the primary outcome: Bone mineral density (BMD)

**FIGURE 1:**
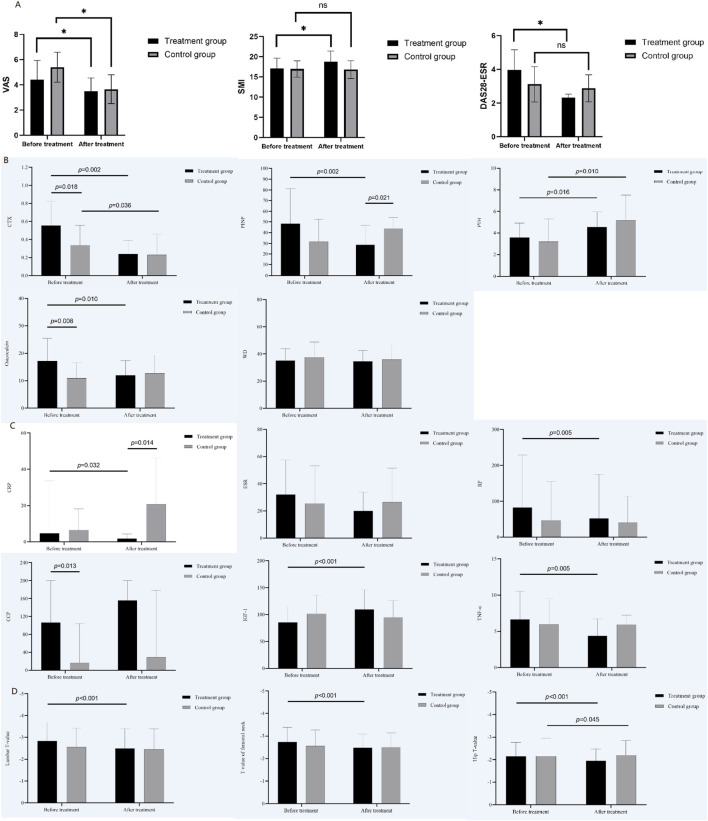
Comparison of clinical outcomes between the treatment group (Group C) and control group (Group D) before and after 12 months of treatment. **(A)** Visual Analog Scale (VAS) scores and Appendicular Skeletal Muscle Mass Index (ASMI); **(B)** Bone turnover markers (CTX, PINP, osteocalcin); **(C)** Inflammatory markers (ESR, CRP, TNF-α, IL-6) and IGF-1; **(D)** Bone mineral density (BMD) T-scores at lumbar spine, femur, and hip. Data are shown as mean ± SD. P < 0.05, P < 0.01 vs. baseline or between groups as indicated.

We first assessed the pre-specified primary outcome, the change in bone mineral density (BMD). After 12 months of treatment, the treatment group (Group C) demonstrated a significant increase in BMD T-scores at the lumbar spine, femur, and hip (all P < 0.01 vs. baseline, Figure 1D). In the control group (Group D), the T-scores at the hip also increased, but to a lesser extent (P < 0.05), while the change in the femur and lumbar spine T-score was not statistically significant (P > 0.05).

### 4.3 Changes in exploratory clinical outcomes

Regarding exploratory clinical measures, Group C showed greater improvement in the DAS28-ESR score compared to Group D (P < 0.01, Table 3). The treatment group also showed improvement in the VAS score and skeletal muscle mass index (SMI) (P < 0.01, Figure 1A), while the difference in VAS score in the control group before and after treatment did not reach statistical significance. The improvement in VAS score was more pronounced in the treatment group.

**TABLE 3:** Comparison of DAS28-ESR and SMI scores before and after treatment in the two groups of patients (x ± s).

Group	Number of cases (n = 40)	DAS28-ESR		SMI (kg/m^2^)	
		Before treatment	After treatment	Before treatment	After treatment
Treatment group	20	3.98 ± 1.18	2.33 ± 0.20^*#^	17.12 ± 2.53	18.77 ± 2.65^*#^
Control group	20	3.11 ± 1.05	2.87 ± 0.80	16.95 ± 2.02	16.80 ± 2.20
t	​	2.441	−2.964	0.235	2.559
P	​	0.019	0.007	0.816	0.015

Compared with pre-treatment in the same group, *P < 0.05; compared with post-treatment in the control group #P < 0.05.

### 4.4 Changes in exploratory biochemical markers

Analysis of exploratory biochemical markers revealed that CTX decreased after treatment in both groups (P < 0.05), with a more substantial decrease observed in the treatment group (Figure 1B). In Group C, PINP decreased and osteocalcin increased, whereas no statistically significant changes in PINP and osteocalcin were observed in the control group.

After treatment in Group C, levels of RF, TNF-α, CRP, and ESR were significantly lower, while IGF-1 levels were significantly higher (P < 0.05). However, CCP and IL-6 levels showed no significant change. In the control group, there were no statistically significant differences in these inflammatory markers or IGF-1 before and after treatment (P > 0.05). Details are shown in Supplementary Figure S1C.

### 4.5 Metabolomics findings

#### 4.5.1 Multivariate analysis and metabolite changes

**FIGURE 2:**
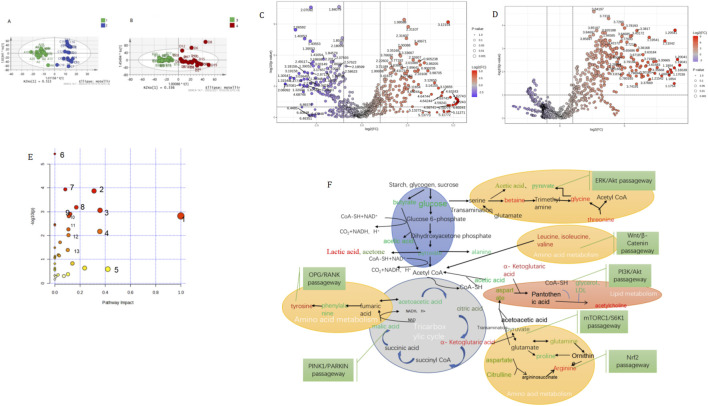
**(A)** OPLS analysis map of treatment group before and after treatment 1: Group C (before treatment in treatment group): 2: Group C (after treatment in treatment group). **(B)** OPLS analysis map of control group before and after treatment: 3: Group D before treatment in control group): 4: Group D (after treatment in control group). **(C)** Volcano plot of the treatment group before and after treatment in Figure 2C. **(D)** The larger the diameter of the origin repents the smaller the P value, the origin on the left represents the metabolite that has increased relatively before treatment and after treatment, and the origin on the right represents the metabolite that has decreased relatively before treatment and after treatment. **(E)** 1. Phenylalanine, tyrptophan biosynthesis; 2. Glycine, serine and threonine metabolism 3. Alanine, aspartate and glutamate metabolism; 4. Phenylalanine metabolism 5. Starch and sucrose metabolism; 6. Valine, leucine and isoleucine biosynthesis; 7. Gloyoxylate and dicarboxylate metabolism; 8. One carbon pool by folate; 9. Arginine biosynthesis; 10. Butanoate metabolism; 11. Pyruvatemetabolism; 12. Gluthione metabolism; 13. Citrate cycle (TCA) cycle. **(F)** Differences id serum metabolomics network before and after RA-OP treatment.

**FIGURE 3:**
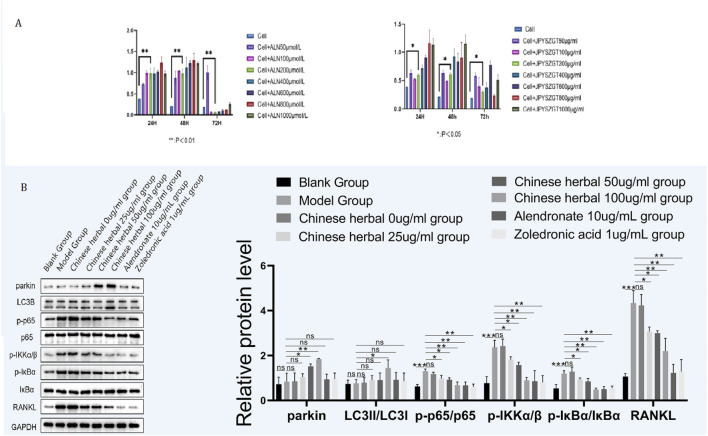
**(A)** Effect of different concentrations of zoledronic acid on the survival rate of RAW264.7. Effect of different concentrations of JPYSZGT on the survival rate of RAW264.7 cells. **(B)** Western blot analysis of protein expression in RAW264.7 macrophages. (Top) Representative blots showing parkin, LC3B, p-p65, p65, p-IKKα/β, p-IκBα, IκBα, RANKL, and GAPDH (loading control) in blank control, model control, JPYSZGT (25 and 50 μg/mL), alendronate, and zoledronic acid groups. (Bottom) Quantitative bar graphs showing relative protein expression levels normalized to GAPDH. Data are shown as mean ± SD. P < 0.05, *P < 0.01 vs. model control group.

Multivariate analysis of serum metabolites showed a clear separation between the treatment group and other groups (Figures 2A,B). OPLS-DA models of the treatment group before and after treatment demonstrated good separation (R2Y = 93.6%, Q2(cum) = 90.1%; Figures 2A,B). Using established criteria (VIP ≥1, P < 0.05) (Li et al., 2025) and following FDR correction (q < 0.2), The treatment group metabolites were identified as significantly altered in the treatment group (Figure 2C; Figure 3; Supplementary Table S1). A parallel analysis of the control group revealed fewer altered metabolites. The control group metabolites after FDR correction; Figure 2D; Supplementary Figure S2; Supplementary Table S2), suggesting a more pronounced metabolic shift in the treatment group.

#### 4.5.2 Potential metabolic pathways

Pathway analysis based on the altered metabolites in the treatment group identified 14 candidate pathways (P < 0.05 or impact >0.1; Table 4 and Figure 2E). These preliminary findings suggest that the intervention may influence pathways related to energy metabolism (e.g., TCA cycle), amino acid metabolism (e.g., valine, leucine, and isoleucine biosynthesis), and lipid metabolism (e.g., glycerophospholipid metabolism), see Figure 2F.

**TABLE 4:** Analysis of major metabolic pathways involved in the serum metabolites of the treatment group before and after treatment.

Pathway name	Total	Hits	Raw p	Impact
Valine, leucine and isoleucine biosynthesis	8	4	3.1826e-06	0
Glycine, serine and threonine metabolism	33	5	0.00011	0.3113
Glyoxylate and dicarboxylate metabolism	32	4	0.00127	0.08333
One carbon pool by folate	26	4	0.00655	0.17047
Phenylalanine, tyrosine and tryptophan biosynthesis	4	2	0.001394	1
Valine, leucine and isoleucine degradation	40	4	0.0029554	0
Pyruvate metabolism	23	3	0.0049273	0.10781
Phenylalanine metabolism	8	2	0.0062588	0.35714
Alanine, aspartate and glutamate metabolism	28	3	0.0086533	0.16186
Arginine biosynthesis	14	2	0.0192	0
Butanoate metabolism	15	2	0.021942	0.11111
Citrate cycle (TCA cycle)	20	2	0.037848	0.10268
Tyrosine metabolism	42	2	0.13927	0.13972
Glycerolipid metabolism	16	1	0.22467	0.23676
Starch and sucrose metabolism	18	1	0.24908	0.4207
Cysteine and methionine metabolism	33	1	0.41004	0.10446

### 4.6 *In vitro* experimental findings

#### 4.6.1 Cell viability and protein expression

Cell viability assays confirmed that JPYSZGT had no toxic effects on RAW264.7 cells under the tested conditions, see Figure 3A. Western blot analysis suggested that JPYSZGT may upregulate proteins related to the PINK1/PARKIN pathway (parkin, LC3B) and downregulate proteins in the NF-κB (p-p65, p-IKKα/β) and OPG/RANK (RANKL) pathways (Figure 3B).

#### 4.6.2 Cytokine secretion

**FIGURE 4:**
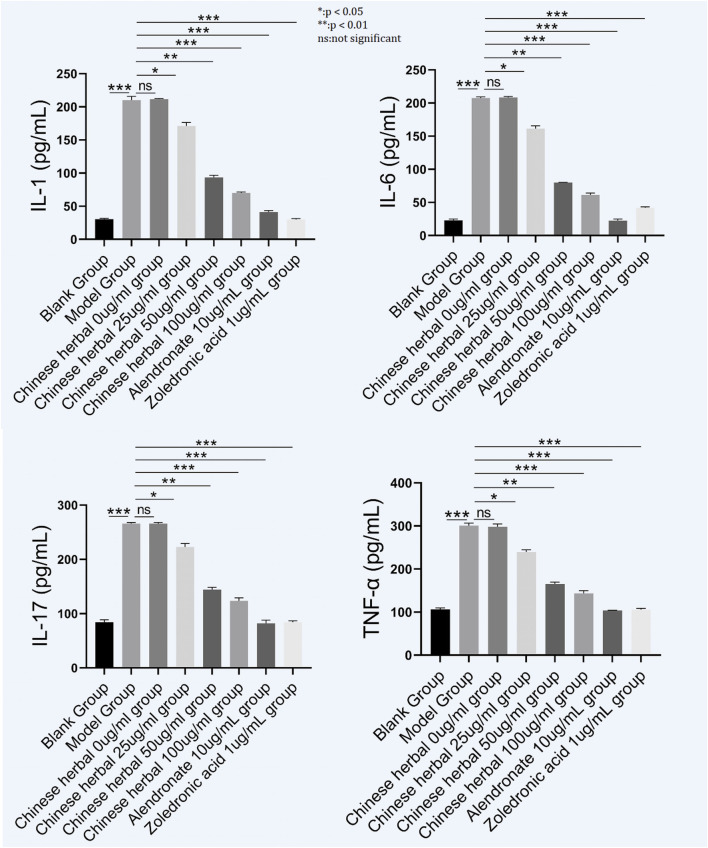
Cytokine secretion profiles in RAW264.7 macrophages treated with JPYSZGT (25 and 50 μg/mL), alendronate, or zoledronic acid. Levels of IL-1, IL-6, IL-17, and TNF-α (pg/mL) were measured by ELISA. Data are shown as mean ± SD. *P < 0.01 vs. model control group; ns: not significant.

JPYSZGT treatment at 25 μg/mL and 50 μg/mL significantly reduced the secretion of pro-inflammatory cytokines TNF-α, IL-1, and IL-6 in the cell culture model compared to the model control group (Figure 4).

### 4.7 Chemical profiling of JPYSZGT preparation

To ensure the quality and batch-to-batch consistency of the JPYSZGT preparation used in this study, we performed UPLC-Q-TOF-MS analysis. The base peak intensity (BPI) chromatograms in both positive and negative ion modes are presented in Supplementary Table S3/Supplementary Figure S1. A total of 152 major peaks were detected. Based on high-resolution mass spectrometry, we successfully tentatively identified 385 key bioactive compounds, which are representative of the constituent herbs. These included Hesperidin, Calycosin, Quercetin and so on. The detailed list of identified compounds, with their retention times and mass spectral data, is provided in Supplementary Table S4. This chemical fingerprint confirms the complex composition of JPYSZGT and provides a reproducible reference for its quality assessment and batch-to-batch consistency.

## 5 Discussion

RA is a chronic inflammatory joint disease and an autoimmune disorder. It affects bone metabolism by causing an imbalance in bone immune regulation, which disrupts normal bone resorption cycles, reduces both local and systemic bone mineral density, and leads to osteoporosis (Yang et al., 2015; Feng et al., 2023). The pathogenesis of RA and sarcopenia-osteoporosis is complex and involves multiple systems. This exploratory research combined a pilot clinical trial, non - targeted metabolomics, and *in vitro* assays to put forward hypotheses about the possible effectiveness and mechanisms of the JPYSZGT formula in treating RA - SO. Our clinical data indicate that JPYSZGT, used as an adjunctive therapy, might boost bone density, increase skeletal muscle mass, and alleviate disease activity, while regulating the serum levels of inflammatory cytokines, bone turnover markers, and IGF - 1. The findings from metabolomics and *in vitro* experiments offer initial clues into the potential mechanical basis behind these clinical observations.

### 5.1 Limitations as an exploratory study

We first acknowledge the limitations of this work. The clinical component was a single-center pilot study with a relatively small sample size, and no formal power calculation was performed beforehand. Consequently, the statistical power and generalizability of our findings are limited; they should therefore be considered preliminary. Also, while the untargeted metabolomic signatures offer insightful clues, they require confirmation via targeted assays in an independent cohort. Moreover, our mechanistic insights are drawn mainly from a single macrophage cell line model. Thus, our conclusions are primarily hypothesis-generating and highlight trends that merit further investigation. Furthermore, no serious adverse events related to JPYSZGT were reported during this study, suggesting a preliminary safety profile. However, the long-term safety and potential drug-herb interactions of JPYSZGT require further evaluation in larger-scale trials with long-term follow-up.

### 5.2 Potential improvement of bone metabolism via regulation of energy metabolism and oxidative stress

Our metabolomics analysis identified several candidate pathways related to energy metabolism, including the citric acid (TCA) cycle, starch and sucrose metabolism, and butyrate metabolism. These findings suggest that JPYSZGT may be associated with a systemic shift in energy utilization patterns. Aerobic oxidation, centered on the TCA cycle, represents the primary pathway for cellular energy production. In rheumatoid arthritis (RA), oxidative stress—defined as an imbalance between pro-oxidant and antioxidant systems—constitutes a key pathological driver of disease progression and bone erosion (Zhang et al., 2022; Tang et al., 2024; Andersen et al., 2025). The chronic autoimmune inflammatory state in RA induces excessive generation of reactive oxygen species (ROS), which not only exacerbates synovial inflammation but also directly disrupts bone metabolic homeostasis (He et al., 2025).

Mechanistically, ROS function as both oxidants and inflammatory mediators that stimulate osteoclastic activity through multiple signaling cascades. Specifically, ROS activate the NF-κB pathway and the NLRP3 inflammasome, both of which are central to inflammatory bone resorption (Shi et al., 2026; Lin et al., 2024; Huang et al., 2026). Furthermore, ROS promote osteoclast differentiation and activation by upregulating the OPG/RANK/RANKL signaling axis, thereby accelerating bone loss (Zhang et al., 2025). These interconnected pathways create a vicious cycle wherein inflammation generates oxidative stress, which in turn amplifies inflammatory responses and bone destruction.

Based on these considerations, we hypothesize that the clinically observed improvement in bone mineral density (BMD) following JPYSZGT treatment may be partially attributable to modulation of energy metabolism and attenuation of oxidative stress. This hypothesis is supported by our *in vitro* findings, which demonstrated that JPYSZGT upregulated proteins involved in the PINK1/PARKIN pathway (parkin and LC3B), a key regulator of mitophagy—the selective autophagic clearance of damaged mitochondria. Enhanced mitophagy represents a potential mechanism for eliminating dysfunctional mitochondria, thereby reducing ROS production and mitigating oxidative stress (Sun et al., 2018). This interpretation aligns with previous studies demonstrating that inhibition of mitophagy exacerbates bone erosion in experimental models of rheumatoid arthritis (Tan et al., 2024). Taken together, our integrated clinical, metabolomic, and *in vitro* data allow us to propose that JPYSZGT may improve bone metabolism in RA-SO patients by enhancing mitochondrial quality control via the PINK1/PARKIN pathway, consequently reducing oxidative stress and its downstream effects on osteoclast activity.

### 5.3 Potential modulation of lipid and amino acid metabolism

Apart from energy metabolism, our data also pointed to potential improvements in glycerophospholipid metabolism, a pathway closely linked to osteoporosis (Song et al., 2023). Along with earlier reports that link PI3K-Akt signaling to glycerophospholipid metabolism and reduced inflammation (Lu et al., 2024), these findings imply that JPYSZGT might also influence bone metabolism by regulating the PI3K/Akt pathway and related metabolic processes. Compared with the control group, glycerol phospholipid metabolism was improved in the treatment group. Our previous study also found that treatment of BMSCs with Chinese medicine compound prescriptions upregulated p-PI3K and p-Akt. These converging lines of evidence suggest that regulation of the PI3K-Akt signaling pathway and its associated glycerol phospholipid metabolism may be another potential avenue through which JPYSZGT influences bone metabolism.

Furthermore, the apparent regulation of multiple amino acid metabolism pathways (e.g., valine, leucine, isoleucine, glycine, serine, threonine, and particularly alanine, aspartate, glutamate, and arginine metabolism) may be significant. The alanine-aspartate-glutamate metabolism pathway is central to energy production and nitrogen disposal. The ornithine metabolic pathway affecting osteoclast differentiation may be related to Nrf2 inhibiting Odc1 expression by reducing intracellular iron (Dong, 2024). Nrf2 is a key regulator of oxidative stress and is regulated by PINK1 and mitochondrial autophagy. We further speculate that improving the ornithine metabolic pathway can enhance the oxidative stress response of osteoclasts via Nrf2 regulation, thereby inhibiting osteoclast activation. Arginine metabolism has been shown to enhance bone mechanical sensitivity (Wang et al., 2024) and promote vascular osteogenesis by improving mitophagy (Shen et al., 2024), which aligns with the findings of this study. The concurrent rise in serum arginine and improvement in skeletal muscle mass index (SMI) supports the idea that JPYSZGT may alleviate sarcopenia partly through modulating arginine metabolism (Hua et al., 2024; Duan et al., 2023).

The apparent regulation of branched-chain amino acids (valine, leucine, isoleucine) may also be significant, as they play important roles in muscle and bone metabolism (Urano et al., 2024; He et al., 2020). Dysfunction in branched-chain amino acids catabolism is involved in the pathogenesis of sarcopenia, and isoleucine, leucine, and valine are positively correlated with muscle mass and muscle strength (Liu et al., 2024). Branched-chain amino acids and IGF-1 are essential for muscle protein synthesis. Moreover, traditional Chinese medicine compound therapy has shown improvement in muscle synthesis, suggesting that it may enhance muscle synthesis by modulating these pathways. Glycine, serine, and threonine metabolism mainly affects bone formation metabolism and is related to the PI3K/Akt pathway (Hao et al., 2022). This is consistent with our previous research results.

### 5.4 Integration with in vitro findings on inflammation and bone resorption

The hypotheses generated from the clinical and metabolomic data find partial support in ourin vitro model. The downregulation of the NF-κB pathway (evidenced by reduced p-p65, p-IKKα/β) and RANKL expression in macrophages, along with reduced secretion of TNF-α and IL-6 (Cavalcanti de Araújo et al., 2024; Zhang et al., 2023; Sun et al., 2020), suggests that JPYSZGT may directly suppress pro-inflammatory macrophage activation and osteoclast differentiation. This anti-inflammatory and potential anti-osteoclastogenic effect, combined with the suggested enhancement of mitochondrial quality control (PINK1/PARKIN), presents a multifaceted, albeit preliminary, mechanistic picture that aligns with the observed clinical trends.

## 6 Conclusion

In conclusion, this exploratory pilot study provides preliminary evidence that the Jianpi Yishen Zhuanggu Tongluo formula, as an adjunctive therapy, may improve bone density and skeletal muscle mass in patients with RA-SO. The ^1^H-NMR metabolomics and *in vitro* data implicate alterations in energy, lipid, and amino acid metabolism, and suggest a potential role for mitophagy (PINK1/PARKIN) and inflammatory (OPG/RANK, NF-κB) pathways in its mechanism of action. These findings position JPYSZGT as a promising candidate for further investigation in larger-scale, definitive trials to validate its efficacy and elucidate the mechanisms proposed herein.

## Data Availability

The original contributions presented in the study are included in the article/Supplementary Material, further inquiries can be directed to the corresponding author.
