# Supplementary material for: Potential efficacy and preliminary mechanistic insights of the Jianpi Yishen Zhuanggu Tongluo formula for rheumatoid arthritis with sarcopenia-osteopenia: an integrated pilot study
Source: Front Pharmacol. 2026 Apr 21;17:1756789. doi: 10.3389/fphar.2026.1756789 (PMC13139347; doi:10.3389/fphar.2026.1756789)
Supplement: Supplementary file 1 [file Supplementaryfile1.docx]

Supplementary Table S1 Changes in serum metabolites before and after treatment in the treatment group

| Name | Chemical shift value | Trend of change | VIP |
| --- | --- | --- | --- |
| N-acetylglucosamine | 2.06 | ↑ | 1.80809 |
| α-ketoglutaric acid | 3.02 | ↑ | 1.86962 |
| glycine | 3.54、3.18 | ↑ | 2.11382 |
| inositol | 3.56 | ↑ | 2.07535 |
| arginine | 3.24 | ↑ | 1.93313 |
| creatine | 3.04 | ↑ | 2.13357 |
| lysine | 1.86 | ↑ | 2.27738 |
| tyrosine | 3.94、6.89 | ↑ | 1.96211 |
| leucine | 1.74、3.73 | ↑ | 1.93756 |
| phosphocholine/choline | 3.22 | ↑ | 1.86439 |
| lactate | 1.31 | ↑ | 2.08739 |
| glutamic acid | 2.46 | ↓ | 1.21484 |
| threonine | 1.32 | ↑ | 2.09698 |
| betaine | 3.26 | ↑ | 1.82595 |
| valine | 2.26 | ↑ | 2.21264 |
| isoleucine | 1.97、3.66 | ↑ | 2.08375 |
| lipoprotein | 0.88 | ↑ | 2.07564 |
| 3-hydroxybutyrate | 2.31 | ↓ | 1.9377 |
| O-acetylglucosamine | 2.14 | ↓ | 1.00945 |
| α-glucose | 3.76 | ↓ | 1.88236 |
| phenylalanine | 3.12、3.98 | ↓ | 1.92889 |
| alanine | 3.77 | ↓ | 1.9462 |
| glycerol | 3.64 | ↓ | 1.50282 |
| citrulline | 3.71 | ↓ | 1.90003 |
| glutamine | 2.45、3.78 | ↓ | 1.507 |
| methionine/histidine | 3.14 | ↓ | 1.62821 |
| malic acid | 3.42 | ↓ | 1.51211 |
| proline | 3.34 | ↓ | 1.8427 |
| acetate | 1.92 | ↓ | 1.50638 |
| acetoacetic acid | 2.22 | ↓ | 1.40715 |
| aspartic acid | 2.67 | ↓ | 1.0414 |
