# Supplementary material for: Potential efficacy and preliminary mechanistic insights of the Jianpi Yishen Zhuanggu Tongluo formula for rheumatoid arthritis with sarcopenia-osteopenia: an integrated pilot study
Source: Front Pharmacol. 2026 Apr 21;17:1756789. doi: 10.3389/fphar.2026.1756789 (PMC13139347; doi:10.3389/fphar.2026.1756789)
Supplement: Supplementary file 2 [file DataSheet1.pdf]

Total ion chromatogram of the sample identification of natural products in JPYSZGT

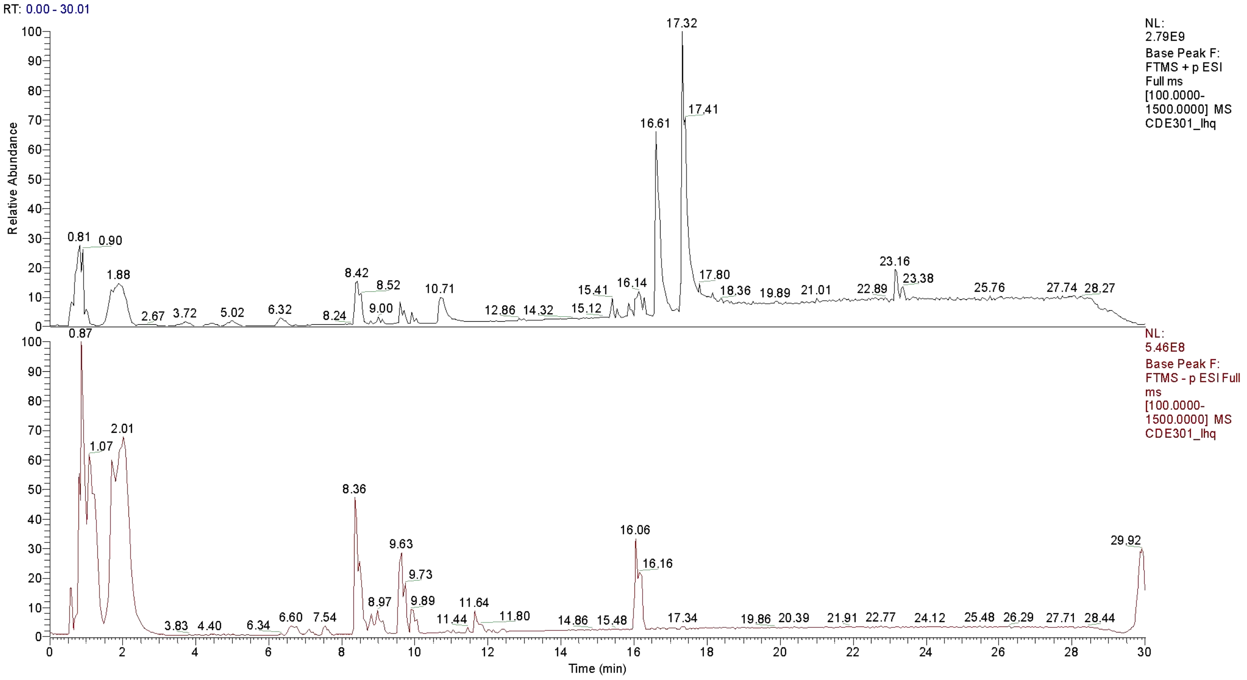

Note: The first column is the total ion chromatogram in positive ion mode, and the second column is the total ion chromatogram in negative ion mode
