# Supplementary material for: Potential efficacy and preliminary mechanistic insights of the Jianpi Yishen Zhuanggu Tongluo formula for rheumatoid arthritis with sarcopenia-osteopenia: an integrated pilot study
Source: Front Pharmacol. 2026 Apr 21;17:1756789. doi: 10.3389/fphar.2026.1756789 (PMC13139347; doi:10.3389/fphar.2026.1756789)
Supplement: Supplementary file 3 [file Supplementaryfile2.docx]

Supplementary Table S2 Changes in serum metabolites before and after treatment in the control group

| Name | Chemical shift value | Trend of change | VIP |
| --- | --- | --- | --- |
| 3-hydroxybutyric acid | 2.31 | ↓ | 1.12546 |
| N-acetylglucosamine | 2.06 | ↓ | 1.1472 |
| O-acetylglucosamine | 2.14 | ↓ | 1.20705 |
| α-ketlutaric acid | 3.02 | ↓ | 1.09943 |
| glycine | 3.54、3.18 | ↓ | 1.14396 |
| glutamine | 2.45、3.78 | ↓ | 1.15364 |
| histidine | 3.14 | ↓ | 1.08401 |
| lysine | 1.86 | ↓ | 1.15774 |
| arginine | 3.24 | ↓ |  |
| tyrosine | 3.94、6.89 | ↓ | 1.05703 |
| leucine | 1.74、3.73 | ↓ | 1.17417 |
| phospholine | 3.22 | ↓ | 1.02381 |
| malic acid | 3.42 | ↓ | 1.12094 |
| valine | 2.26 | ↓ | 1.1698 |
| acetate | 1.92 | ↓ | 1.06585 |
| acetoacetic acid | 2.22 | ↓ | 1.16755 |
| isoleucine | 1.97、3.66 | ↓ | 1.18085 |
| tryptophan | 7.19 | ↓ | 1.08801 |
| glutamic | 2.46 | ↓ | 1.15 |
